# An adaptive image enhancement method for a recirculating aquaculture system

**DOI:** 10.1038/s41598-017-06538-9

**Published:** 2017-07-24

**Authors:** Chao Zhou, Xinting Yang, Baihai Zhang, Kai Lin, Daming Xu, Qiang Guo, Chuanheng Sun

**Affiliations:** 1 Beijing Research Center for Information Technology in Agriculture, Beijing, 100097 China; 2 National Engineering Research Center for Information Technology in Agriculture, Beijing, 100097 China; 3 National Engineering Laboratory for Agri-product Quality Traceability, Beijing, 100097 China; 4 School of Automation, Beijing Institute of Technology, Beijing, 100081 China

## Abstract

Due to the low and uneven illumination that is typical of a recirculating aquaculture system (RAS), visible and near infrared (NIR) images collected from RASs always have low brightness and contrast. To resolve this issue, this paper proposes an image enhancement method based on the Multi-Scale Retinex (MSR) algorithm and a greyscale nonlinear transformation. First, the images are processed using the MSR algorithm to eliminate the influence of low and uneven illumination. Then, the normalized incomplete Beta function is used to perform a greyscale nonlinear transformation. The function’s optimal parameters (*α* and *β*) are automatically selected by the particle swarm optimization (PSO) algorithm based on an image contrast measurement function. This adaptive image enhancement method is compared with other classic enhancement methods. The results show that the proposed method greatly improves the image contrast and highlights dark areas, which is helpful during further analysis of these images.

## Introduction

A recirculating aquaculture system (RAS) is a highly efficient artificially controlled system that provides a suitable growth environment for fish through a variety of technologies^1, 2^. As a low-cost and non-contact method, many scholars have studied the application of computer vision technology to RASs because it is important for guiding production and decision-making^3, 4^. Practice has shown that near infrared and vision-based computer vision is quite suitable for image acquisition and fish monitoring in an RAS^5, 6^. However, because of the insufficient and uneven illumination in commercial fish farms and because most species of fish can change their skin colour to adapt to the ambient colour^7–9^, the captured images always have both low contrast and very bright backgrounds. As a result, detailed information can easily be lost^10, 11^, which makes it difficult to recognize the intended targets and distinguish them from other fish^12^.

Many studies have proposed methods of enhancing the images to improve the contrast. By simulating the visual perception of the human eye, the Multi-Scale Retinex (MSR) algorithm effectively improves the image contrast and reveals details previously obscured by shadows or light^13, 14^. Therefore, the MSR is often used to process medical images, remote sensing images, foggy images and low-contrast images; furthermore, the MSR is used in image enhancement and other applications^15^. However, the images become excessively bright after processing with the MSR algorithm^16, 17^. To improve the uneven background in the particle images and enhance their contrast, the dynamic range of the image grey levels must be increased^18–20^. Numerous grey transform enhancement methods exist and can be broadly divided into two categories: spatial domain methods and frequency domain methods^21^. Among these methods, the commonly used classical transform methods include linear enhancement (LE), histogram equalization (HE), wavelet transform (WT), and contrast limited adaptive histogram equalization(CLAHE)^21^. However, in most visible and infrared images, the traditional HE method produces an unsatisfactory outcome, as the background noise with the typical grey levels is amplified and the detailed information is constrained by the typical grey levels^22–24^. Although the LE method is relatively simple, its enhancement effect is greatly influenced by parameter selection. The WT method achieves image enhancement through the attenuation process of the high-frequency wavelet coefficients. The main drawback of this method is that it cannot enhance all of the parts of the image; therefore, it is difficult for the algorithm to achieve adaptive image enhancement^25–27^. There are also many improved algorithms to enhance the image contrast, including intelligent optimization algorithms such as the artificial bee colony algorithm (ABC) and the particle swarm optimization algorithm (PSO). In addition, fuzzy and genetic algorithms (GAs) have been used in the image contrast enhancement process^28–33^. However, the illumination in the RAS changes constantly, and the objects being monitored are typically uncontrollable and move rapidly. When using the above algorithms to process the resulting images, the parameters for these algorithms cannot be adjusted automatically to match changes in field conditions nor can they completely solve the problem of low contrast caused by a lack of light, unevenness and fish behaviour. Therefore, to meet real-time processing requirements, an adaptive image enhancement method is needed that can adjust the parameters for intelligent algorithms automatically according to changes in the environment and the monitored objects.

On the basis of simulating the commercial-scale fish farm environment, the current study proposes a near infrared and visible image enhancement method to improve the image contrast in the RAS. The image enhancement was performed by uneven illumination corrections and nonlinear transforms based on the MSR algorithm and greyscale nonlinear transformation. Furthermore, possible factors that could influence the enhancement results were taken into account. In addition, the optional parameters of the incomplete Beta function were selected by the PSO algorithm. To assess the reliability of our method, it was also compared with the results of other methods. The purpose of this study was to build a potential method to enhance the contrast of the image in RAS, and we aim to provide accurate and consistent segmentation for subsequent image processing.

## Materials and Methods

### Experimental System

**Figure 1:**
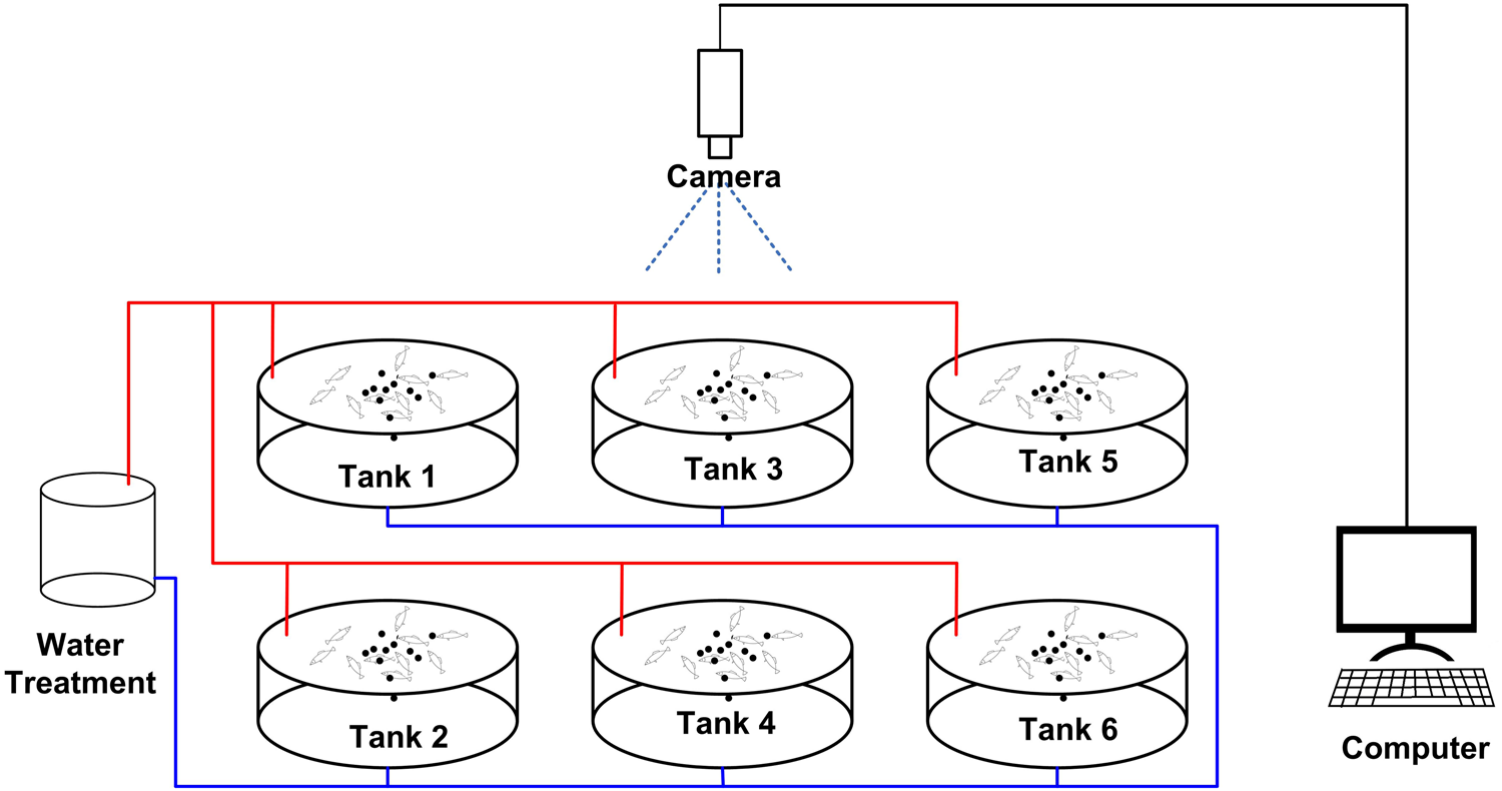
The experimental system.

The experiment was conducted in the RAS laboratory of the Xiaotangshan National Experiment Station for Precision Agriculture, Beijing, China. The RAS system was assembled for the image acquisition and analysis that we previously described (see Fig. 1)^5^. The system has six tanks, and each tank has a diameter of 1.5 m and a water depth of 1 m. A near infrared (NIR) industrial camera (AVT Mako G-223B, Stadtroda, Germany) was fixed above the water surface, and the distance was determined to be 1.5 m during tests of the system. This value permitted monitoring of most of the water volume of the tank. The camera had a bit depth of 8/12 and a resolution of 2048 × 1088. The images of the tank were captured sequentially at the rate of one frame per second, and visible and near infrared images of 400–1000 nm were collected. The raw output data stream from the camera was converted to BMP files by software we developed through the software packages provided by AVT. A computer was connected to the camera to achieve real-time image acquisition and processing. The image processing was implemented in MATLAB® (The MathWorks Inc., Natick, MA, USA).

### MSR image enhancement algorithm

To obtain a high-quality segmentation effect, it is necessary to enhance the images to improve the contrast between the target and the background. In this study, Retinex theory was used to enhance the images. The basic principle of Retinex theory is that the reflection and luminance components are used to decompose the image. MSR is one of the most commonly used algorithms in the domain of image enhancement. The MSR method improves the greyscale and contrast of the images and has attracted wide attention from researchers^34^. Indeed, this method can maintain image fidelity while increasing the dynamic range of the image compression^15^. The Multi-Scale Retinex in the logarithm domain can be expressed as shown in Equation (1) ^16, 35^.

1 $${\rm{R}}(x,y)=\sum _{k=1}^{M}{\omega }_{k}\{\mathrm{log}\,[I(x,y)]-\,\mathrm{log}\,[F(x,y,{c}_{k})\,\ast \,I(x,y)]\}$$

Where I(x, y) is the original image and R(x, y) is the reflected image. Here, $F(x,y,{c}_{k})$ is the Gaussian surround function, which can be expressed as in Equation (2)^15^:

2 $$F(x,y,{c}_{k})=M\,\exp [-\frac{{x}^{2}+{y}^{2}}{{{c}_{k}}^{2}}]$$

In Equations (1) and (2), M is the number of scales and ${\omega }_{{\rm{k}}}$ is the weight factor. In general, M and ${\omega }_{{\rm{k}}}$ are typically set to 3 and 1/3, respectively. Additionally, c_k_ is the scale parameter. Whereas smaller values of c_k_ imply better image details, larger values of c_k_ imply better consistency of the image’s colour. Generally, when the values of c_k_ are set to 15, 80 and 250, the Retinex enhancement algorithm has a better dynamic range and better colour reproduction characteristics, resulting in a better visual effect^15^.

Mathematically, the MSR algorithm subtracts the convolution value of the Gaussian function and original image from the original image in the logarithm space. The algorithm actually subtracts the parts of the lighting that change. After applying the MSR algorithm, the details in dark areas are highlighted, and the influence of uneven illumination on the image is improved. However, the processed image is bright, and the contrast is still low. To improve this situation, it is necessary to use the corresponding transformation function for greyscale transformation.

### Nonlinear transform enhancement

**Figure 2:**
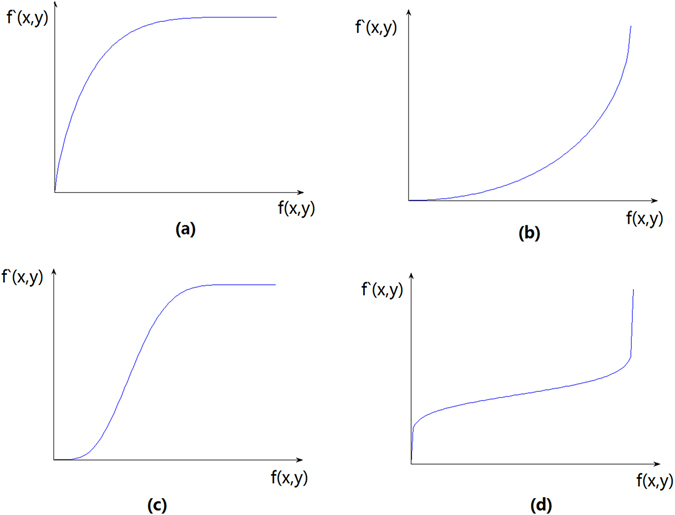
Four types of nonlinear transformation for greyscale image enhancement: (**a**) a transform stretching dark regions; (**b**) a transform stretching lighter regions; (**c**) a transform stretching the middle and compressing the two ends; (**d**) a transform compressing the middle and stretching the two ends.

Different transform functions are used for the different cases of dark, bright or greyscale over concentration; the corresponding transfer functions are shown in Fig. 2. The horizontal coordinate f(x, y) is the greyscale of the original image, and the vertical coordinate f′(x, y) is the processed greyscale^29, 36^.

To enhance the greyscale image, Tubbs proposed an incomplete Beta function^37^ that can completely replace the four types of transform functions for image enhancement. The incomplete Beta function can be expressed as shown in Equation (3):

3 $$F(u)={B}^{-1}(\alpha ,\beta )\times {\int }_{0}^{u}{t}^{\alpha -1}{(1-t)}^{\beta -1}dt$$

where *B*(*α, β*) is the Beta function given by Equation (4):

4 $$B(\alpha ,\beta )={\int }_{0}^{1}{t}^{\alpha -1}{(1-t)}^{\beta -1}dt$$

In Equation (4), 0 < *α*, *β* < 10, and 0 < *μ* < 1.The function is determined by the parameters *α* and *β*, whose values determine the shape of the transformation curve.

### Particle swarm optimization

The *α* and *β* parameters of the incomplete Beta function determine the type of transformation. This study used the PSO algorithm to automatically select the optimal parameter values for *α* and *β* that maximize the image enhancement.

The PSO algorithm, which was first proposed by Kennedy and Eberhart in 1995^38^, has been applied to numerous areas, such as pattern recognition, multi-objective optimization and signal processing^39–41^. The algorithm finds the optimal result by delivering information and information sharing. It is a high-performance parallel search algorithm that performs a global search strategy based on a swarm of particles^42^.

Assume that N is the size of the swarm, M is the search space dimension, ${{\rm{X}}}_{{\rm{i}}}({x}_{i1},{x}_{i2},\cdots ,{x}_{iM})$ is the spatial position of the *i*-th particle, ${{\rm{V}}}_{{\rm{i}}}({v}_{i1},{v}_{i2},\cdots ,{v}_{iM})$ is the velocity, ${P}_{i}({p}_{i1},{p}_{i2},\cdots ,{p}_{iM})$ is the optimal position of the space, and ${P}_{g}({p}_{g1},{p}_{g2},\cdots ,{p}_{gM})$ is the optimal position of all of the particles travelling in the current swarm. In each iteration, each particle’s velocity and spatial position are updated according to Equations (5) and (6), respectively^43^:

5 $${v}_{im}^{k+1}={\omega }_{k}{v}_{m}^{k}+{c}_{1}\times rand()\times ({p}_{im}-{x}_{im}^{k})/{\rm{\Delta }}t+{c}_{2}\times rand()\times ({p}_{gm}-{x}_{im}^{k})/{\rm{\Delta }}t$$

6 $${x}_{im}^{k+1}={x}_{im}^{k}+{v}_{im}^{k}{\rm{\Delta }}t$$

Where m is the *m*-th dimension of the search space (1 ≤ m ≤ M), k is the number of iterations, c_1_ and c_2_ are acceleration constants, the rand() function returns a random number between 0 and 1, ∆t is usually given in time units, and *ω* is the inertia weight.

This study used the contrast measurement function as a fitness function to quantitatively evaluate the enhancement provided by the PSO. This fitness function is shown in Equation (7)^44^:

7 $$Fitness=\frac{1}{N\times M}\sum _{x=1}^{M}\sum _{y=1}^{N}f\prime\prime\prime {}^{2}(x,y)-[\frac{1}{N\times M}\sum _{x=1}^{M}\sum _{y=1}^{N}f\prime\prime\prime (x,y){]}^{2}$$

where M and N are the width and height of the image, respectively, and *i* is the number of a particle. In addition, f(x, y) is the original grey value of the pixel (x, y), and $f\prime\prime\prime (x,y)$ is the grey value of the pixel after enhancement. A larger image fitness value indicates a more uniform greyscale distribution and a higher contrast, which results in better image quality.

### Adaptive image enhancement method

The detailed computational steps of the adaptive image enhancement method using the MSR and PSO algorithms are as follows:

Step 1: Calculate the original image grey value I(x, y);

Step 2: Process the original image by the MSR algorithm according to Equation (1) to obtain f(x, y);

Step 3: Perform the normalized transformation for each pixel using Equation (8):

8 $$f^{\prime} (x,y)=(f(x,y)-{L}_{\min })/({L}_{\max }-{L}_{\min })$$

Where f′(x, y) is the normalized greyscale value of the pixel (x, y), and L_max_ and L_min_ are the maximum and minimum grey values of the original image, respectively.

Step 4: Apply the PSO algorithm to select the parameter values for *α* and *β* of the incomplete Beta function.

Step 5: According to the *α* and *β* values selected in the previous step, each pixel of the normalized image is then enhanced using Equation (9),

9 $$f^{\prime\prime} (x,y)=F(f^{\prime} (x,y))$$

where F is the incomplete Beta function in Equation (2), and f′(x, y) is the normalized value of pixel (x, y).

Step6: According to the image grey value range, perform the inverse transform of each pixel using Equation (10) to obtain the resulting image.

10 $$f\prime\prime\prime (x,y)=(L{^{\prime} }_{\max }-{L^{\prime} }_{\min })f^{\prime\prime} (x,y)+L{^{\prime} }_{\min }$$

where L′_max_ and L′_min_ are the maximum and minimum grey values of the resulting image, respectively. Note that these values depend on the number of bits in the image; for an 8-bit image, L′_max_ = 255 and L′_min_ = 0.

### Image quality evaluation

An evaluation of the effect of the image enhancement can be conducted from two viewpoints: the subjective perception and quantitative analysis. The subjective perception of the human eye is the simplest, most direct, and most effective way to evaluate an image and is widely used. For images with large differences and variations, reaching a broad consensus is easy. However, the subjective perception also has some limitations. For images with less obvious differences, humans cannot easily judge which of the differences requires a quantitative evaluation. There are many indices with which one can quantify the contrast^45^. In this paper, the contrast, mean square error (MSE), peak signal-to-noise ratio (PSNR) and information entropy were used to evaluate the image enhancement effects. The contrast can be obtained from Equation (7), and the MSE, PSNR and information entropy are respectively defined as follows^46–49^:

11 $$MSE=\frac{1}{MN}\sum _{x}\sum _{y}{[I(x,y)-\hat{I}(x,y)]}^{2}$$

12 $${PSNR}=10\,{\mathrm{log}}_{10}\frac{{m}^{2}}{MSE}$$

13 $$H(x)=-\sum _{i=1}^{n}p({x}_{i})\mathrm{log}(p({x}_{i}))$$

In the above equations, M × N is the size of the original image I(x,y), $\hat{I}(x,y)$ represents the processed image, m represents the maximum value an image pixel can achieve(e.g., for an 8-bit greyscale image, m = 255), *x*_*i*_ is the greyscale value of the *i*-th image pixel, and p(x_i_) is the occurrence probability of ${x}_{i}(i=1,2,\cdots ,{x}_{n})$, where $0\le p({x}_{i})\le 1$ and $\sum _{i=1}^{n}p({x}_{i})=1$. Note that p(x_i_) can be obtained from the greyscale histogram of the image. Among these measurements, the contrast, MSE and PSNR reflect the differences between the enhanced image and the original image. Generally, a smaller MSE value and larger PSNR and contrast values indicate a better processing effect, and a higher information entropy indicates that the image contains a higher degree and larger quantity of information.

### Data Availability

The datasets generated during and/or analysed during the current study are available from the corresponding author on reasonable request.

## Results and Discussion

### Image enhancement results analysis

**Figure 3:**
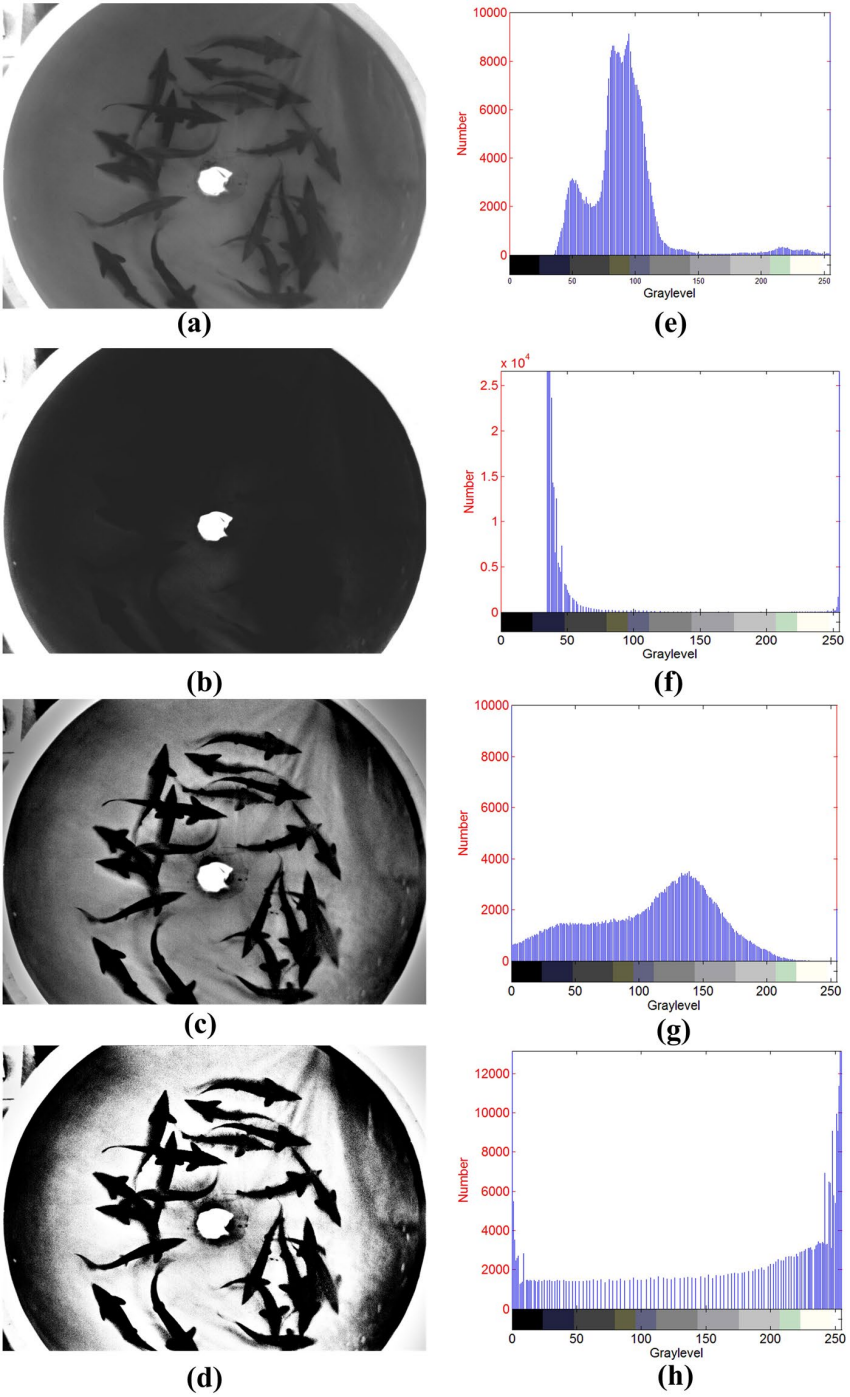
Image enhancement effect: (**a**) original image; (**b**) nonlinear transformation of Fig. 3a; (**c**) the original image enhanced by the MSR algorithm; (**d**) proposed method; (**e**) histogram of Fig. 3a; (**f**) histogram of Fig. 3b; (**g**) histogram of Fig. 3c;(**h**) histogram of Fig. 3d.

In this study, the original image was first enhanced by the MSR algorithm. In the original image (Fig. 3a),the contrast is low and the visual effects are poor. Moreover, the greyscale histogram (Fig. 3e) shows that the greyscale distribution is not smooth and uniform. After processing with the MSR algorithm, the visual effect of the enhanced image (Fig. 3c) has been improved. Although the greyscale histogram in Fig. 3g shows that the greyscale distribution has become smoother and more continuous, it is still too concentrated. Therefore, the image greyscale must be stretched and transformed.

### Nonlinear transform enhancement results analysis

During the greyscale nonlinear transform enhancement process, the image is transformed using the nonlinear Beta function whose optimal parameters are selected by the PSO algorithm. The swarm size is N = 10, the maximum number of iterations is T_max_ = 100, the acceleration constants are c_1_ = c_2_ = 2,and the maximum and minimum inertia weights are *ω*_*max*_ = 0.9 and *ω*_*min*_ = 0.4, respectively. Lastly, the maximum velocity is *v*_*max*_ = 5^50–52^.

Figure 3b was obtained when the original image (Fig. 3a) was directly processed using a nonlinear transformation (without the MSR algorithm). Figure 3f shows that the grey distribution is too concentrated in the dark area. In some cases, either the MSR algorithm or the nonlinear transformation can be used to achieve good results. However, when dealing with images in the RAS, applying these techniques may cause the grey distribution to become discontinuous or excessively concentrated.

**Figure 4:**
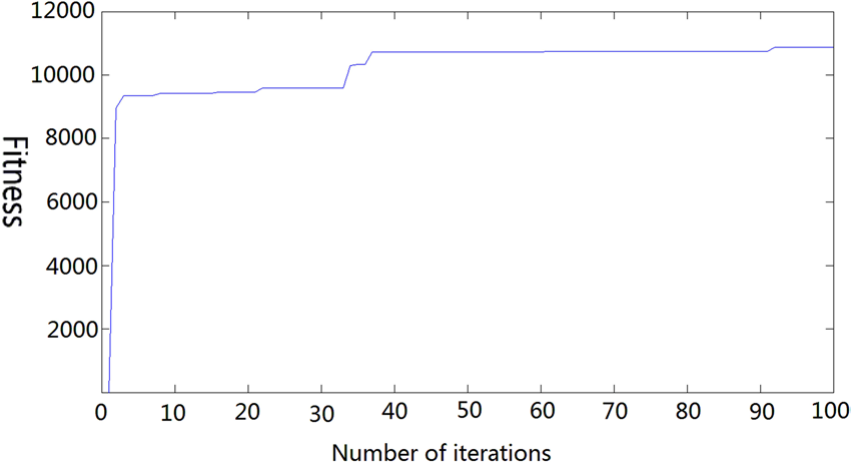
PSO algorithm optimization process.

**Figure 5:**
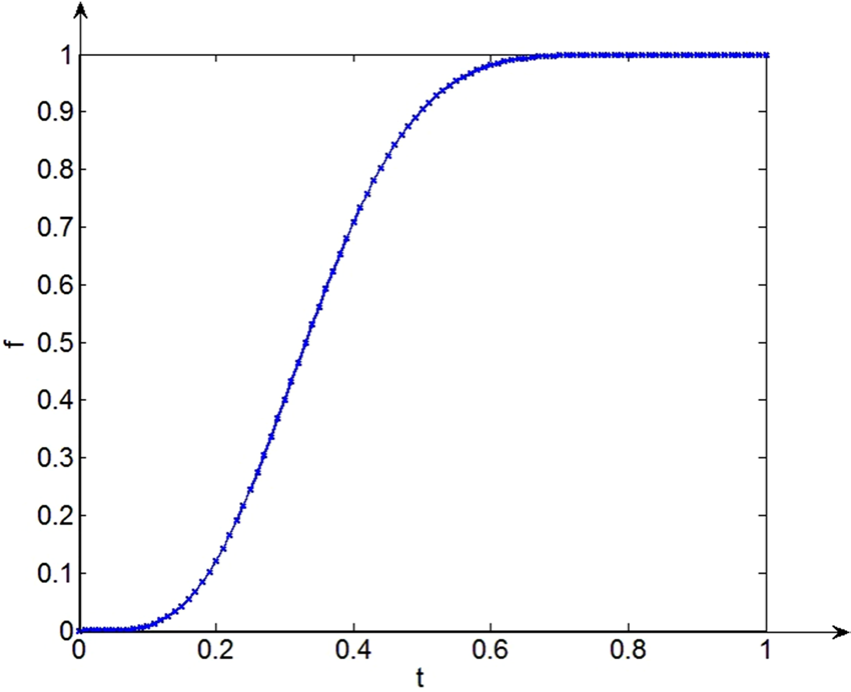
Transformation function curve.

To solve this problem, the MSR algorithm is used to smooth the image; then, the image is transformed by the incomplete Beta function. As shown in Fig. 4, after 40 iterations, the fitness function’s value is already very close to the optimal value. After 92 iterations, the function remains stable until the optimal value of 10867 is found. The position of the corresponding particle group is *α* = 5.09, *β* = 9.97, and the function is given by Equation (14). Finally, the transformed picture (Fig. 3d) is obtained. Figure 5 shows the function curve, which conforms to type (c) in Fig. 2. After stretching the greyscale, the picture conforms more convincingly to people’s visual expectations. Compared to the original image, the clarity and contrast of the fish, tank and other details are obviously improved. The greyscale distribution is also more uniform and continuous (Fig. 3h), and the distribution of the light and dark grey areas is more reasonable. Thus, the overall visual effect of the enhanced image is better and the robustness is enhanced.

14 $$F(u)={B}^{-1}(5.09,9.97)\times {\int }_{0}^{u}{t}^{4.09}{(1-t)}^{8.97}dt$$

### Enhanced image quality evaluation and comparison

The qualities of the enhanced images were evaluated objectively and quantitatively by calculating the evaluation indices. As shown in Table 1, the image quality evaluation index of the original image (Fig. 3a), the image enhanced by the MSR algorithm (Fig. 3c), the image directly enhanced by the nonlinear transformation (Fig. 3b) and the image enhanced by the proposed method (Fig. 3d) were all calculated. Compared to the original image, the contrasts of all of the enhancement methods have improved, and the contrast of the proposed method reaches the highest value. This finding indirectly validates the above conclusions.

**Table 1:** Quality evaluation of MSR and the nonlinear transform.

	Contrast	PSNR	MSE	Information entropy
Original image	2807			5.3364
MSR	3642	27.2926	121.2890	6.0822
PSO	4590	24.7255	219.0436	5.4549
MSRPSO	10867	38.3670	54.7059	6.5552

**Figure 6:**
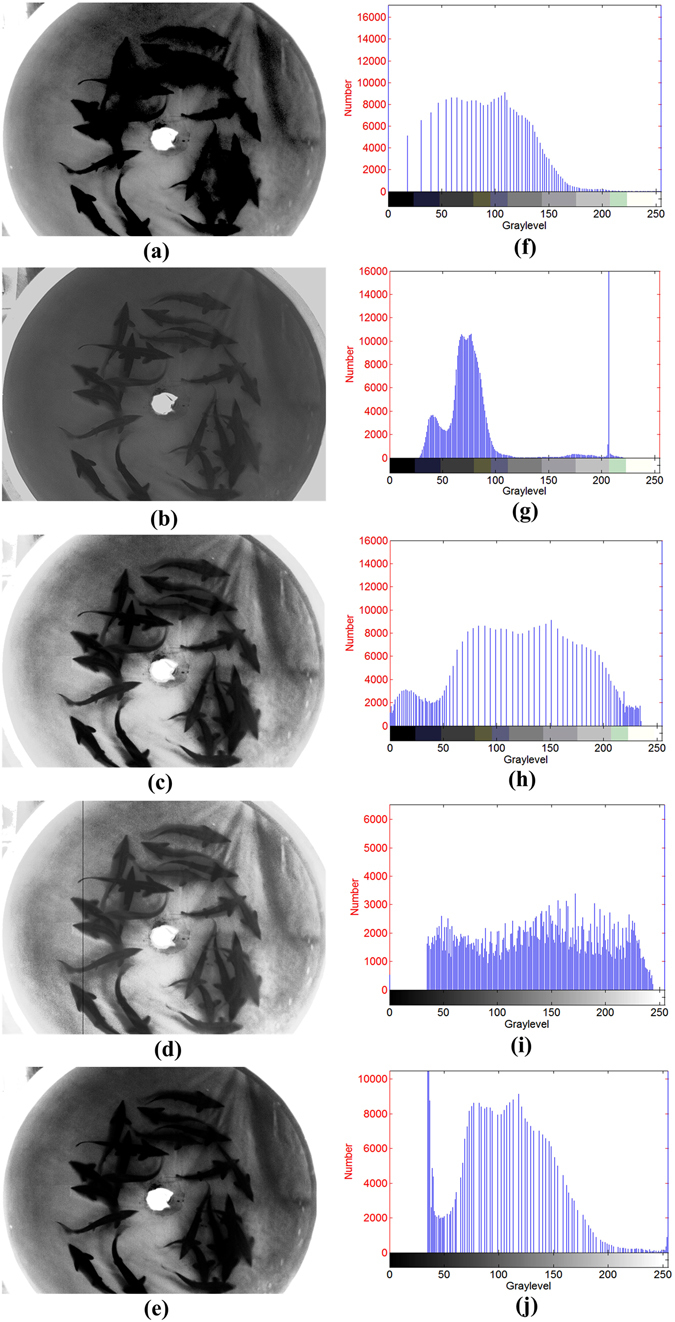
Results of other enhancement methods: (**a**) LE; (**b**) WT; (**c**) HE; (**d**)CLAHE; (**e**)GA-based;(**f**)histogram of Fig. 6a; (**g**) histogram of Fig. 6b; (**h**) histogram of Fig. 6c; (**i**) histogram of Fig. 6d; (**j**) histogram of Fig. 6e.

To demonstrate the performance of the proposed algorithm, we implemented some related methods and compared their results with those of the proposed method. This comparison was performed in terms of the contrast and detail enhancement of the proposed method. The contrast enhancement methods used for comparison purposes are LE, HE, WT, CLAHE, and genetic algorithm-based enhancement (GA-based). The enhancement result for Tank 1 is shown in Fig. 6.

From a human perspective, all of the enhancement methods improve the contrast; however, when compared with the original images(Fig. 3a and Fig. 3e),the linear enhancement method causes the grey values to become concentrated (Fig. 6a and d). The contrast improvement by the wavelet transform method is not obvious (Fig. 6b and e), the histogram equalization and contrast limited adaptive histogram equalization method lose some details (Fig. 6c and f, Fig. 6d and i), and the genetic-algorithm-based enhancement causes greyscale concentration. The proposed method achieves the best visual effect (Fig. 3d and h) as the resulting enhanced image is clear and its information is rich.

Table 2 lists the contrast, PSNR, MSE and information entropy of each of the two images captured from two tanks and calculated by Equation (7). After applying six enhancement methods, the contrast in both images increases. The method proposed in this paper has the smallest MSE and the highest contrast and PSNR, which indicates that the proposed method has the best enhancement effect. The objective and quantitative evaluations are consistent with the subjective visual evaluation. Still, the time for each iteration was 1.85 s, compared with the GA-based (2.17 s), CLAHE (1.84), LE(0.15 s), WT (0.48 s), and HE (0.34 s). Although this processing time is not the best, it is acceptable because the speed at which fish swim is not as rapid as the industrial field. In addition, the processing time can be accelerated based on the actual application with methods such as appropriate utilization of the parallel processing, hardware acceleration, and structuring element decomposition^18^.

**Table 2:** Quality Evaluation Index.

		Contrast	PSNR	MSE	Information entropy
**Tank 1**	**Original image**	2807			5.3364
	**LE**	5776	28.2126	98.1340	6.0573
	**WT**	2885	24.6684	221.9430	5.9144
	**HE**	5586	29.8839	66.7870	6.1327
	**CLAHE**	4086	44.0190	62.5774	7.4554
	**GA-based**	5595	27.1226	126.1293	5.8317
	**Proposed method**	10867	38.3670	54.7059	6.5552
**Tank 2**	**Original image**	3263			5.4176
	**LE**	5963	32.2271	91.9378	6.3668
	**WT**	3313	24.2247	245.8159	5.7637
	**HE**	5769	28.5214	67.3982	6.2149
	**CLAHE**	4034	29.5656	71.8654	7.0822
	**GA-based**	3422	35.4719	58.4457	5.9113
	**Proposed method**	11529	39.8506	38.3008	6.6063

### Comparisons to different species

**Figure 7:**
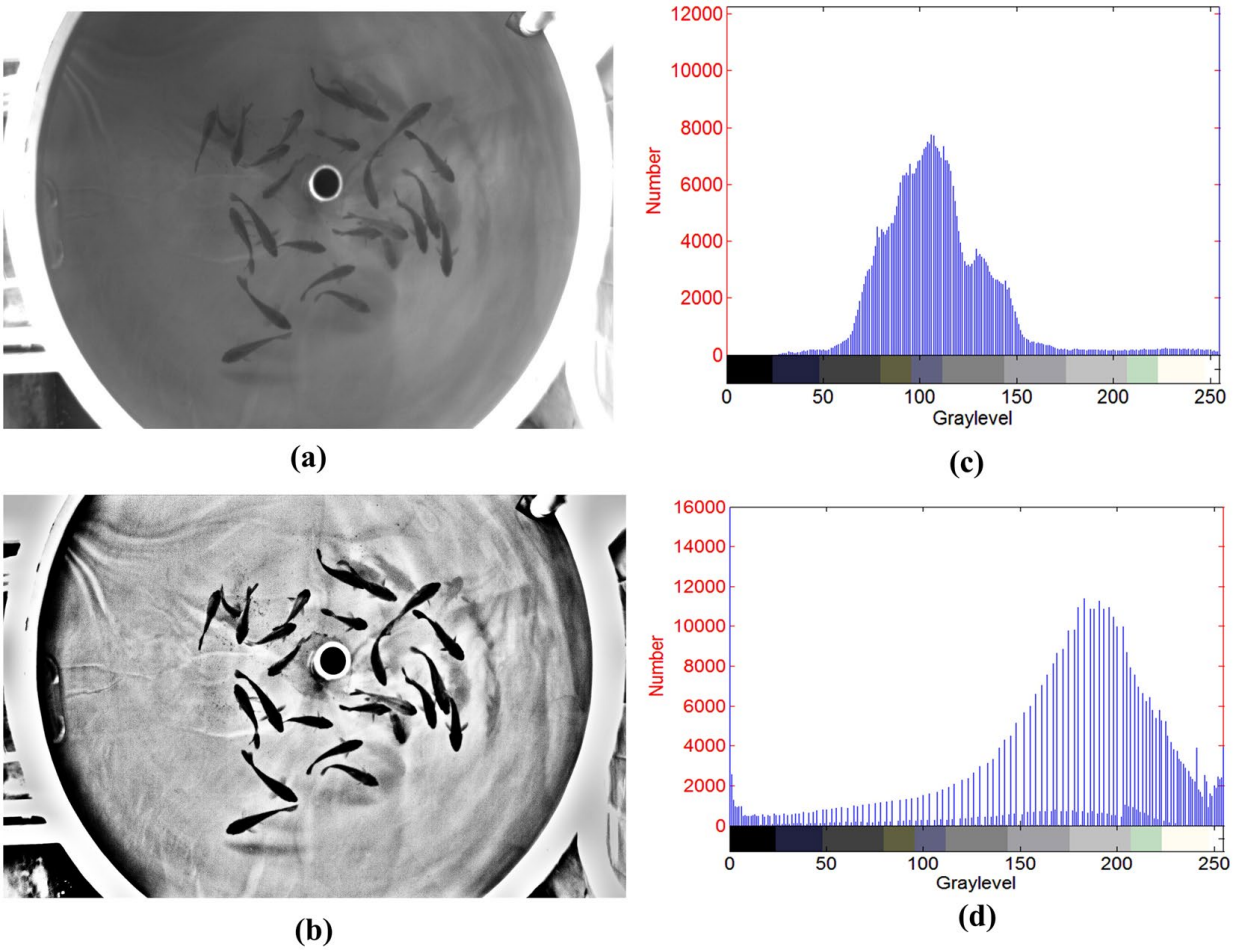
Image enhancement results of carp: (**a**) original image of carp; (**b**) enhancement results; (**c**) histogram of Fig. 7a;(**d**) histogram of Fig. 7b.

**Figure 8:**
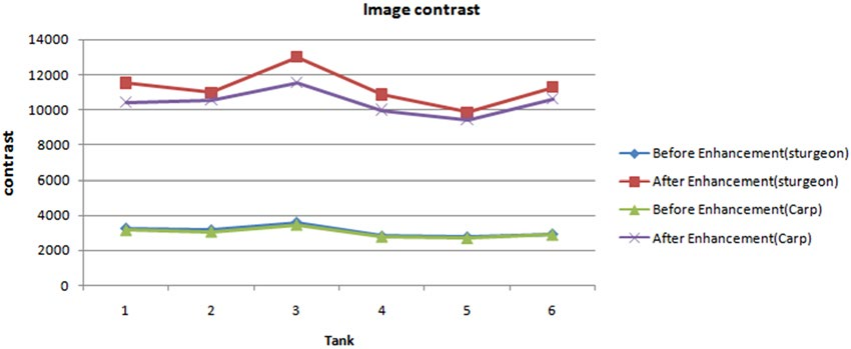
Image contrast of the two species.

**Figure 9:**
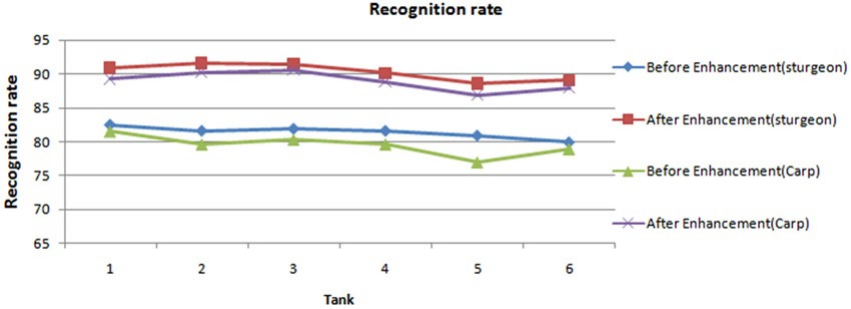
Recognition rates of carp and sturgeon.

The recognition rates achieved during later image processing were used to compare the performance of the proposed method with different fish species. Images of carp *(Cyprinus carpio var. specularis)* and sturgeon *(Acipenser baeri Brandt)* from six tanks were collected and enhanced. We used methods of image processing and recognition-rate calculation that were described in Zhou, *et al*. ^5^ and Pautsina, *et al*. ^6^, and the concatenated images were segmented using a watershed algorithm^53^. Finally, the recognition rates of carp and sturgeon in six tanks were calculated. As shown in Fig. 7, after the enhanced method, the details of the carp’s image become clear, the contrast is significantly improved and formerly hidden details are revealed. Figure 8 and Fig. 9, respectively, show that the contrasts and recognition rates of the images of the two species were improved. Indeed, compared with the original images of sturgeon and carp, the average recognition rates were increased by 7.9% and 9.5%, and the average contrasts were improved by factors of 3.65 and 3.47, respectively. This result demonstrates that the method provides a substantial enhancement to images of carp and sturgeon.

However, we found that both before and after enhancement, the recognition rates and contrasts of the images of carp are always lower than those of the images of sturgeon. After analysis, we determined that because the carp used in the experiment are non-scaly fish whose skin colour is dark, they reflect near infrared light more weakly than sturgeon. Consequently, the enhancement effect is lower, and this method has a better effect on images of scaly fish than on those of non-scaly fish. Although the results showed that errors still occur, the enhanced images perform significantly better than the images without enhancement. Considering the errors caused by the other steps, these results are acceptable.

## Conclusion

In this paper, we demonstrated that the proposed method can effectively improve the contrast of images using the MSR algorithm and a nonlinear transform whose optimization parameters were selected automatically using the PSO algorithm. A comparison of the proposed method with other classical enhancement algorithms showed that the proposed method effectively reduces the influence of low and uneven illumination on subsequent recognition results. The proposed method enhances the contrast of the image and provides a good foundation for subsequent image processing. Therefore, it could function as an important and feasible technique for image pre-processing in the future and is an important step in processing the computer vision images used in RASs to achieve accurate and automatic target identification. However, this method needs more study to find the optimal solutions to improve the contrast of non-scaly fish before it can be used for image processing and target recognition and the computing speed still needs to be improved.
